# Photobiomodulation for Alzheimer’s disease: photoelectric coupling effect on attenuating Aβ neurotoxicity

**DOI:** 10.1007/s10103-022-03692-z

**Published:** 2023-01-12

**Authors:** Zixi Tian, Panpan Wang, Kai Huang, Jie Yu, Mange Zhang, Yanming Liu, Hang Zhao, Beilei Zhu, Xuerong Huang, Zhiqian Tong

**Affiliations:** 1 Key Laboratory of Alzheimer’s Disease of Zhejiang Province, Institute of Aging, Oujiang Laboratory, School of Mental Health, Wenzhou Medical University, Wenzhou, 325035 China; 2 Department Neurology, Wenzhou Medical University Affiliated Hospital 3, Wenzhou, 325200 China

**Keywords:** Alzheimer’s disease, Amyloid-beta, Endogenous formaldehyde, Near-infrared light, Photoelectric coupling effect, Photobiomodulation

## Abstract

Alzheimer’s disease (AD) and dementia are the most worrying health problems faced by people globally today. Although the pathological features of AD consisting of amyloid-beta (Aβ) plaques in the extracellular space (ECS) and intracellular tau tangles are well established, the developed medicines targeting these two proteins have not obtained the expected clinical effects. Photobiomodulation (PBM) describes the therapeutic use of red light (RL) or near-infrared light (NIR) to serve as a noninvasive neuroprotective strategy for brain diseases. The present review discusses the mechanisms of the photoelectric coupling effect (light energy-induced special electronic transition-related alterations in protein structure) of PBM on reducing Aβ toxicity. On the one hand, RL or NIR can directly disassemble Aβ in vitro and in vivo. On the other hand, formaldehyde (FA)-inhibited catalase (CAT) and H_2_O_2_-inactived formaldehyde dehydrogenase (FDH) are formed a vicious circle in AD; however, light energy not only activates FDH to degrade excessive FA (which crosslinks Aβ monomer to form Aβ oligomers and senile plaques) but also sensitizes CAT to reduce hydrogen peroxide levels (H_2_O_2_, which can facilitate Aβ aggregation and enhance FA generation). In addition, it also activates mitochondrial cytochrome-c to produce ATP in the neurons. Clinical trials of phototherapeutics or oral coenzyme Q10 have shown positive effects in AD patients. Hence, a promising strategy combined PBM with nanopacked Q10 has been proposed to apply for treating AD.

## Introduction

Alzheimer’s disease (AD) is the most common form of dementia affecting more than 50 million people globally in 2018. With the disease burden expected to exceed 152 million by 2050 according to World Alzheimer Report 2018 [1]. People live longer nowadays. Most countries have entered the ageing society, and ageing is the main cause of dementia. The disability rate of AD is high, and the patients lose the ability to live independently in the terminal stage, bringing a heavy economic burden on the family and society. It has been one of the main diseases affecting the sustainable development of the global economy.

The amyloid-beta (Aβ) deposition in the brain extracellular space (ECS) to appearance senile plaques (SP) and tau hyperphosphorylation to form neurofibrillary tangles (NFTs) are the two typical pathological characteristics of AD [2, 3]. However, in the past decades, antibodies, vaccines, or small molecule drugs aimed against the production, aggregation, and clearance of Aβ and tau have not achieved ideal clinical efficacy [4]. Aβ deposition in the brain ECS to form SP has been found in AD over 100 years [5], and there are numerous hypotheses about what endogenous factors induce Aβ aggregation; however, no one has been confirmed so far.

Recent studies have revealed that ageing induces a marked elevation in endogenous formaldehyde (FA) levels in the brains [6], and urine FA concentrations were positively correlated with cognitive decline in aged humans [7]. In particular, excessive FA in the brains is considered to be a critical trigger of Aβ aggregation and cognitive dysfunction [8–10]. For example, the results of in vitro experiments showed that FA at pathological concentration crosslinked Aβ monomer into dimer, trimer, oligomers, and fibrils. The data of in vivo experiments indicated that Aβ-binding with FA dehydrogenase (FDH) led to endogenous FA accumulation in the brains [11]; especially, injection of FA promoted Aβ oligomerization and SP formation in the brain of APP/PS1 mice [9, 12]. Notably, injection of FA can directly promote tau hyperphosphorylation and NFTs formation [13]. In addition, injection of FA at a pathological concentration (which was detected in APP/PS1 mice) can mimic ageing-induced memory impairments in healthy adult male mice [10]. These data support the viewpoint that accumulated endogenous FA is closely related to the occurrence and development of AD.

## Photobiomodulation for Alzheimer’s disease

How to find out an effective therapeutical method for AD is a worldwide difficulty. Owing to the failure of drug developments to treat AD in the world, more and more researchers start to pay attention to nondrug therapy. The safe and noninvasive nondrug methods to improve cognitive functions and alleviate mental disorders in AD patients become urgently needed in the global. PBM may be a promising strategy for AD treatment.

### The development of phototherapeutics

PBM is a method to treat diseases and enhance the recovery of the body by using the warm effect, photochemical effect, photobiological regulation, and other characteristics of sunlight or artificial light, including infrared, ultraviolet, visible light, and laser [14]. According to the choice of spectrum, it can be divided into the following: full spectrum irradiation, such as natural light illumination, bright light therapy (BLT), and monochromatic light irradiation, such as red, blue, and compound light. For example, the laser diode also called injection laser diode stands for light amplification by stimulated emission of radiation. This electronic device transforms the electrical energy provided by the input source into the beam of light. It has the characteristics of high brightness (high energy density), good directivity (directional radiation and small divergence angle), pure monochromaticity (the purest light color and single light wave frequency), and good coherence [15]. If the laser cannot directly cause irreversible damage in clinical trials, it is a weak laser, which is mainly used in physiotherapy and named low-level laser therapy (LLLT) with less than 100 ~ 200 mW. It can produce benign biological stimulation, responses, and photochemical effects, so as to regulate the functions of the immune system, nervous system, blood circulation system, and tissue metabolism [16, 17].

### Patterns of photobiomodulation

There are three possible patterns for the current study for PBM in AD: (1) retinal pathway. For example, the 40 Hz white light scintillator via eyes can attenuate the pathological characteristics of AD mice [18]. (2) Nonretinal access includes body surface exposure, endovascular irradiation, and nasal exposure. (3) Direct irradiation pathway includes helmet-type transcranial illumination [19]. BLT and LLLT are the most investigated in the clinical application of AD. Among them, LLLT has better curative effects on AD, and near-infrared light of ~ 1000 nm is mostly used [20], but the “thermal effect” of this wavelength is obvious. Its side effects including mild mania, migraine, eye fatigue, nausea, and agitation, have been observed in clinical trials [21]. In 2015, a phototherapeutic device with red light at shorter wavelength (630 ± 20 nm) had been developed in China [22], which has been proved to have positive clinical effects, less thermal side effects, and better security than NIR.

### Photobiomodulation improves cognitive function in animals and patients

Multiple methods of PBM have been found to improve the cognitive function of AD patients. For example, clinical BLT therapy (1000 lx) can ameliorate cognitive disorders in AD patients [23, 24]. Intravascular red light treatment for 20–40 min can improve cognition [25]. Transcranial treatment combined with intranasal near-infrared irradiation at 810 nm alleviates cognitive decline [26]. Near-infrared light (1060–1080 nm) has been found to improve cognitive performance [27]. All-day bright light combined with melatonin can improve cognitive function and sleep quality in patients [28]. The results of animal models showed that near-infrared light at 1070 nm rescues memory deficits in AD model mice [29]. Some studies of LLLT on biological cells have been carried out; particularly, LLLT at 632.8 nm irradiation on AD model mice attenuates memory decline [30].

## Photobiomodulation with high-energy red light

The above-mentioned Chinese phototherapeutic device is composed of helmet and belt with 630-nm RL. The helmet is used to irradiate Aβ-deposited brain region (hippocampus, prefrontal, parietal, and occipital lobe) of AD patients. The belt is carried out to illuminate live (a main detoxification organ) to activate FDH for degrading FA.

There were two reasons why 630-nm wavelength was selected. First, RL at 630 nm has been found to reduce Aβ-mediated SP in brain ECS and decrease intracellular AβO in APP/PS1 mice. It also activates FDH to degrade formaldehyde, thereby reducing Aβ deposition in brain ECS and rescuing the drainage of the interstitial fluid (ISF) [11]. Second, RL at 630 nm has few thermal effects but can penetrate the skull [11, 31, 32]. Although RL or NIR with longer wavelengths can penetrate the skull more easily, light at over 650 nm has a “heating effect” [31–33], which most likely induces clinical side effects, such as headache, insomnia, and stroke [34].

## Red light disassembles Aβ fibrils via photoelectric coupling effects

**Fig. 1:**
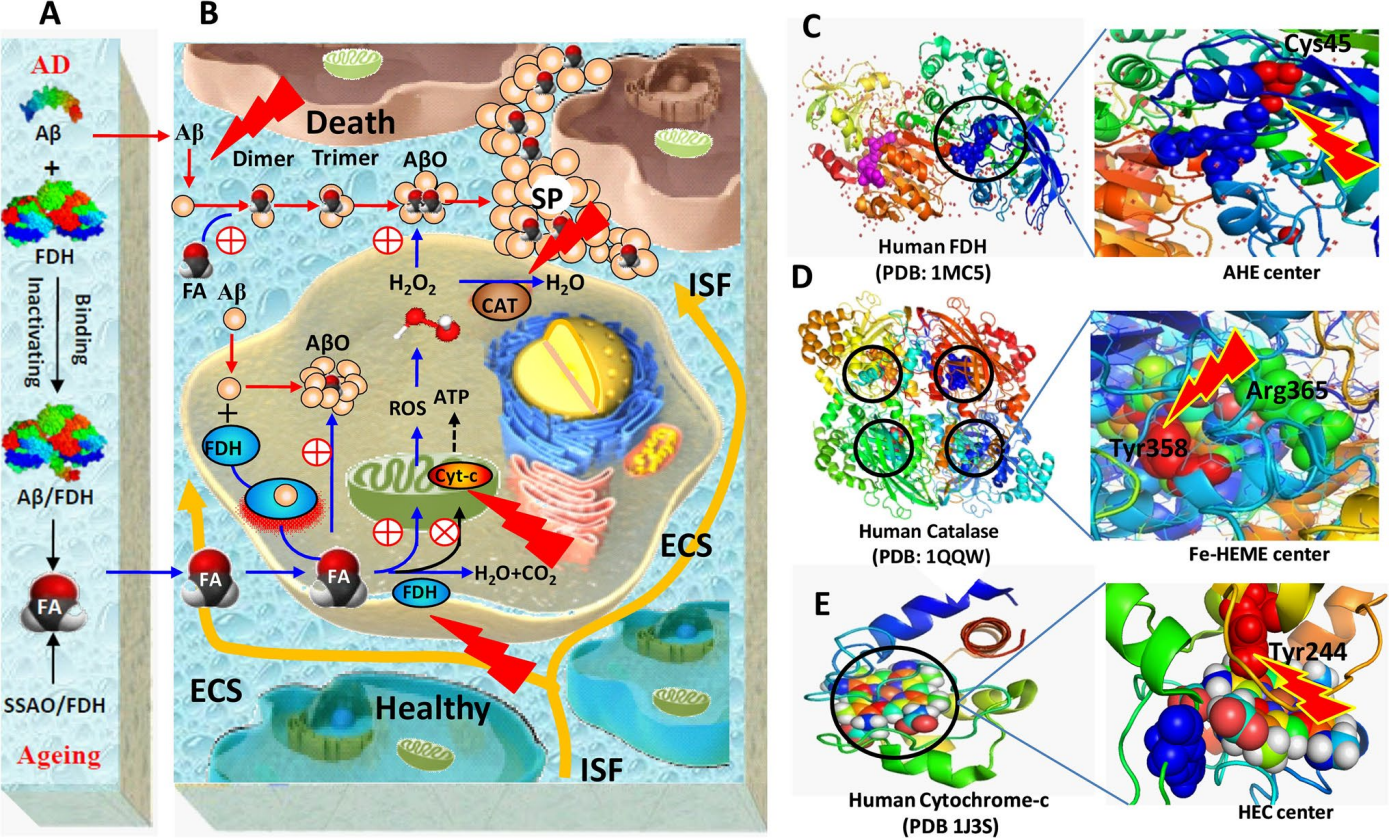
Model of photoelectric coupling effects of photobiomodulation on Alzheimer’s disease. (**A**) FA accumulation derived from Aβ-inactivating FDH and expression imbalance of FA-generating and FA-degradating enzyme (SSAO and FDH). (**B**) Photobiomodulation with RL or NIR on FA-promoted Aβ neurotoxicity. Red symbols (flash): red or near-infrared light illumination. (**C**) Model of laser-activating human FDH. (**D**) Model of laser-activating human catalase. (**E**) Model of laser-activating human cytochrome-c. AD, Alzheimer’s disease; AHE: C_11_H_19_N_3_O_7_S; Aβ, amyloid-beta; AβO, Aβ oligomers; ATP, adenosine triphosphate; CAT, catalase; Cyt-c, cytochrome-c; Cys, cysteine; ECS: extracellular space (diameter: 38 ~ 64 nm); FA, formaldehyde; FDH, formaldehyde dehydrogenase; Fe, ferrum; Fe-HEME: the Fe(III) heme; HEC: heme C; ISF, interstitial fluid; ROS, reactive oxygen species; SSAO, semicarbazide-sensitive amine oxidase; SP, senile plaques; Tyr, tyrosine

A previous study has shown that there are changes in the secondary structure of Aβ40 or the complex of Aβ40-C60 examined by using circular dichroism (CD) after the incubation of 5 days at 37 °C in the purified protein solutions with blue or red photoirradiation, respectively [35]. Different PBM techniques have been established to reduce Aβ self-assembly [36–39]. Recent study has found that FA can bind with 28^th^ lysine residue of Aβ42 monomer and enhance Aβ assembly; however, RL at 630 nm irradiating Aβ solution markedly reduced the formation of Aβ fibrils in vitro [9]. It also decreased Aβ-mediated SP deposition in ECS and intracellular AβO in APP/PS1 mice [11] (Fig. 1A, B).

### RL directly disassembles FA-crosslinking Aβ

#### Formaldehyde is a critical trigger of Aβ aggregation

Notably, a clinical investigation has shown that blood FA levels were gradually elevated in the aged human [6]. Unexpectedly, excessive FA can crosslink Aβ monomer to form dimer, trimer, oligomers, and fibrils in vitro. In AD, Aβ-binding with FA dehydrogenase (FDH) causes FA accumulation in the brains [11]. FA also elicits Aβ oligomerization and SP formation in the brain of APP/PS1 mice [9, 12].

#### Metabolical pathways of endogenous formaldehyde

Gaseous FA is the simplest small organic molecule that first appeared in the early evolution of the earth, containing C, H, and O elements at the same time [40]. Unexpectedly, endogenous FA exists in the cells of all living things [41]. FA production and degradation enzymes have been shown to regulate the balance of endogenous FA levels in the organism of eukaryotes [8, 42], which can avoid its neurotoxic and cytotoxicity [43, 44].

*Formaldehyde-generating enzyme* FA is endogenously generated by the following enzymes: sarcosine dehydrogenase (SARDH), TET methylcytosine dioxygenase 1 (TET1), lysine specific demethylase 1 (LSD1), endoplasmic reticulum demethylase, semicarbazide-sensitive amine oxidase (SSAO), and mitochondrial cytochrome P450 enzyme.

*Formaldehyde-degrading enzyme* endogenous FA is mainly degraded by glutathione-dependent FA dehydrogenase (FDH, also known as ADH3), alcohol dehydrogenase 1 (ADH1), and GSH-independent aldehyde dehydrogenase 2 (ALDH2). Then, S-methyl GSH dehydrogenase, glyoxalase, and catalase (CAT) can also degrade FA.

#### Physiological and pathological functions of endogenous formaldehyde

Exogenous air pollutant-gaseous FA indeed induces cognitive impairments in a survey in 75,322 participants [45]. Recently, endogenous FA has been proven to dually regulate memory formation. Under physiological condition, learning activity and high-frequent stimulations can elicit a transient elevation in the active FA levels, which are generated in the mitochondria of hippocampal neurons via SARDH; especially, this elevated active FA can activate NMDA-receptor and enhance memory formation [46]. However, FA overload suppresses NMDA-receptor by crosslinking NR1 and NR2B, which inhibits memory formation [46].

Clinical survey showed that blood FA levels were gradually elevated in the aged human and a marked increase in the age of 70 [6]. This suggests that endogenous FA was accumulated during the ageing process, and 70 may be the key point of memory decline. In a clinical investigation in 604 elderly and 517 dementia patients, uric FA concentration was positively correlated with cognitive decline than age-matched controls [10, 47]. Remarkably, the imbalance of expression and activity of FA-generating enzyme-SSAO and degrading enzyme-FDH is the critical reason for FA accumulation during ageing process [47, 48]. In APP/PS1 mice, there is a vicious circle between FA accumulation induced by Aβ-inactivated FDH and FA-promoted Aβ oligomerization intracellularly and fibrillation extracellularly (Fig. 1A, B), which leads to irreversible memory decline [8, 9, 12]. Hence, scavenging of FA contributes to the treatment of AD.

#### RL reduces the effects of FA-crosslinking Aβ

The irradiation of RL at 630 nm can change the secondary-helical structure of Aβ; thus, it reduces the formation of fibrils [11]. The light energy at a special wavelength can couple with the special chemical bonds of biological proteins, causing electron transitions, inducing protein disassembly or enzyme activation [49]. RL destructing the dimer of FA-crosslinked Aβ is the direct cause of Aβ disassembly [9, 11].

### RL at 630 nm indirectly disassembles Aβ fibrils

#### Photoelectric coupling activates FDH to degrade FA

A previous study found that FA is the critical factor to form Aβ dimer and quickly form trimers, oligomers, and fibrils, while the addition of FA scavengers reduces the formation of Aβ fibers in vitro [9]. Age-related FA accumulation in the brains can enhance the formation of SP and NFTs [47, 50], which leads to AD occurrence [9, 12]. However, 630-nm RL can photocouple the thiol group (Cys-45), which binds with AHE (C_11_H_19_N_3_O_7_S) of FDH, to promote FA degradation [49]. FDH contains the common structure Zn^2+^-thiolate catalytic center. Loss of catalytic Zn^2+^ or mutation of Cys45 binding with catalytic Zn^2+^ of FDH (also named GSNOR) leads to the FDH inactivation [51]. Cys45 residues binding with catalytic Zn^2+^ were oxidized by H_2_O_2_ and associated with a release in Zn^2+^ and loss activity of hFDH [49] (Fig. 1C), thus reducing intracellular Aβ oligomerization and extracellular SP deposition in ECS [11].

#### Photoelectric coupling activates CAT to degrade H_2_O_2_

During the ageing process, H_2_O_2_ was gradually accumulated in the brain. H_2_O_2_ not only increases the accumulation of FA [52] but also directly promotes the aggregation of Aβ to form SP [53]. However, 630-nm RL can activate catalase (CAT) by photoelectric coupling to tyrosine (Tyr) residue and promote H_2_O_2_ degradation. Tyr-358 is the catalytic center of CAT and participates in the oxidation–reduction reaction of the Fe(III) heme (Fe-HEME). Consistently, FA has a spontaneous chemical reaction with Tyr directly (6, 52). Thus, excess FA most likely binds to Tyr residue and inactive catalase [49] (Fig. 1D); thus reducing the aggregation of Aβ.

#### Photoelectric coupling activates cyt-c to increase ATP generation

It has been found that the photons produced by NIR or RL can pass through the bone and be absorbed by the chromo group of the mitochondria of neurons, photoelectric coupling to cytochrome-c oxidase (cyt-c). For example, 630-nm laser light can activate cyt-c, which also contains an active center, Tyr244 binding to heme; and the redox status of heme in cyt-c responded to red laser light [54]; Tyr244 participates in the oxidation–reduction reaction of heme C (HEC, C_34_H_34_FeN_4_O_4,_) [55]. Herein, the active center of Tyr-358-binding heme in human CAT may be similar to the model of cyt-c (Fig. 1E). RL or NIR has been proven to directly increase the generation of cell ATP [56, 57]. Irradiation of rats with 660-nm RL stimulates a dose-dependent increase in oxygen consumption and ATP generation in the cerebral cortex by enhancing cyt-c activity [58]. The irradiation of NIR at 808 nm also improves the generation of ATP in the cerebral cortex [59]. This may be the possible reason that the impaired neurons could be rescued by PBM in AD.

#### LLLT accelerates Aβ clearance in the brain and liver

New research suggests that exposure to a light flickering at 40 Hz can promote gamma brain wave activity through the photic entrainment phenomenon [18]. Because the suprachiasmatic nucleus is linked to the light dark cycle [60], robust light–dark patterns are critical for controlling circadian Aβ clearance from the brain to the liver in AD model mice and humans [61]. Hence, this is another possible mechanism that RL or NIL can disassemble Aβ in vivo [62], which accelerates brain-liver Aβ kinetics [63].

In a word, aging-associated FA metabolism disorders and Aβ-inhibited FDH lead to endogenous FA accumulation in the brains; in turn, excessive FA crosslinks the Aβ monomer to oligomerization, tau hyperphosphorylation to form NFTs in the cytoplasm, and SP formation in ECS. FA also induces ROS generation, and H_2_O_2_ promotes Aβ aggregation. Meanwhile, FA-inhibited cytochrome-c reduces ATP generation in the mitochondria, while PBM alleviates Aβ neurotoxicity by reducing Aβ assembly intracellularly and extracellularly; especially, it activates FDH, CAT, and Cyt-c, respectively (Fig. 1C, D). In addition, PBM can reduce the levels of inflammation factors and oxidative stress [11], increase neurogenesis and synaptogenesis [64], improve mitochondrial activity and ATP generation [65], and accelerate blood flow [66]; subsequently, it contributes to the treatment of AD.

## Effect of photobiomodulation on inflammatory factors in AD

It has found that RL or NIR reduces SP numbers [8, 9] and alleviates cognitive deficits in AD transgenic mice by disassembling Aβ, [11]; however, multiple effects of PBM may occur to ameliorate the course of dementia; for example, it can reduce inflammatory factors in AD. NIR at 1070 nm can reduce perivascular microglia and rescue memory deficits in AD model mice [29]. The 40 Hz white light scintillator can increase microglia colocalization with Aβ to scavenge SP [18]. Light also can attenuate Aβ-induced superoxide and inflammation in astrocytes [67–69].

Remarkably, acute exogenous FA exposure induces early Alzheimer-like changes in mouse [70]. It can mimic inflammatory reaction during atherogenesis [71]. FA stimulates the release of inflammation factors, for example, IL-1, IL-6, and TNF-α [72–74]. However, LLLT reduces inflammation factors caused by gaseous FA exposure [75]. Red light at 630 nm can decrease the levels of IL-1β and TNF-α in AD transgenic mice [11]. Hence, the PBM-reduced inflammatory factor contributes to the treatment of AD.

## Prospects of photobiomodulation for AD

Over the past century, the world has been faced with problems including a high incidence and poor drug efficacy for AD. How to reduce Aβ toxicity through noninvasive physical therapy is an emerging field in research of AD. Noninvasive physical therapy has become a new direction that develops rapidly at present. However, it also remains some problems on laser therapy such as acting slowly and poor compliance due to the thermal effect of light. Therefore, it is urgent to investigate the molecular mechanism of more effective laser to disassemble Aβ, decrease the thermal effect of light, improve the penetration rate of skull, and enhance clinical efficacy.

Notably, excessive FA inhibits Cyt-c activity and reduces coenzyme Q10 levels in the mitochondria, which finally induces neuron death; however, Q10 (an endogenous FA scavenger) can degrade FA, reduce Aβ oligomers and SP, and rescue memory functions in APP/PS1 mice [9]. Assessing serum Q10 levels has been proposed to predict the development of dementia [76], and AD [77]. Encouragingly, an enhanced water-soluble nano-Q10 can improve cognitive functions in AD model mice [78]. A combination of PBM and nano-Q10 for treating AD has been found to be more therapeutically effective than one of these methods used alone [79, 80]. This is based on the facts that this kind of combination treatment has positive effects on reducing oxidative stress and neuroinflammation in a depression model mice [81] and a model of transient global brain ischemia [79]. It also can prevent Aβ assembly in AD model mice and alleviate PD-like behaviors in PD model mice [82, 83, 84]. Hence, the combination of these two methods to accelerate ISF drainage will contribute to Aβ clearance and drug delivery in AD patients [8] (Fig. 1B).

Low-level laser therapy (LLLT) is not carcinogenic and teratogenic to animal tissues. Through multiple molecular signaling pathways, it regulates cell functions, improves cell survival, promotes neural stem cell proliferation, and, subsequently, alleviates the pathological characteristics of AD model mice. Some preliminary clinical investigations have found that LLLT as a noninvasive adjuvant treatment is a promising therapeutic strategy for AD patients. It is worth looking forward to the fact that the thermal effect of laser light may be ameliorated by regulating the pulse frequency, duty cycle, and light intensity.

## Data Availability

It was not added.

## Code availability

It was not applicable.
